# Culturally Diverse Perceptions of EEG and Neurofeedback Research and How to Address Them to Reduce Sampling Bias

**DOI:** 10.1111/psyp.70077

**Published:** 2025-05-30

**Authors:** Hedwig Eisenbarth, Chelsea D'Cruz, Joseph A. Bulbulia, Bohemian Thanni

**Affiliations:** ^1^ Victoria University of Wellington Wellington New Zealand

**Keywords:** attitudes, bias, interest, neurofeedback, neurophysiology

## Abstract

Little is known about whether cultural norms affect participation in Electroencephalography (EEG) research in general and in the applied context of EEG‐based neurofeedback for emotion regulation training. As EEG administration requires direct contact with the head, this might interfere with cultural norms regarding the appropriateness of touching the head, and thereby prohibit individuals from taking part in such studies. However, the exclusion of participants given their cultural background limits generalization. To better understand a variety of cultural views, we investigated the perception of and attitudes towards EEG and neurofeedback across a culturally diverse group from Aotearoa New Zealand (*N* = 181). Descriptive and content analyses of online survey responses across all participants showed that most participants were not sure what EEG was or were unsure about its function. Knowledge about the neurofeedback methods was also minimal. Participants had helpful suggestions for making the research environment more comfortable. However, using neurofeedback for emotion regulation training was seen critically. Even within this research‐keen, largely NZ European group, knowledge of EEG and neurofeedback was patchy —a gap that probably dampens participation by other cultural groups. Providing clear information upfront, creating a welcoming study environment, and letting participants choose the technician's gender should broaden the inclusiveness of future neuroscience research.

## Abstract 2

EEG research might be subject to selection bias related to culture, but we do not know what concerns participants could have about being involved in EEG neurofeedback research. Our findings allow us to derive a series of suggestions for research practice to make EEG research more accessible, including for recruitment information and procedural aspects during setup and recording.

## 1 Introduction

Electroencephalography (EEG) is a method of measuring brain activity that the general population rarely experiences. Not only are EEG assessments, which involve placing sensors on the head, a novel experience for many, but the act of touching the head holds cultural or religious significance for some groups, potentially leading to cultural bias in research samples. Such biases may inhibit generalizations and external validity. In addition, the use of EEG for brain training (neurofeedback), for example, in the context of emotion regulation, might be even less commonly known. Therefore, it is important to understand individuals' cultural values that might interfere with the use of EEG measurement and how individuals respond to the opportunity to take part in general and intervention research that involves EEG. However, surprisingly, neuroscientific research involving EEG has taken little notice of selection bias related to culture. Moreover, we are unaware of any previous research that has investigated the concerns participants themselves might have about being involved in general EEG research or EEG‐based neurofeedback research for emotion regulation training. To address this gap, we investigate attitudes towards and perception of EEG research across a culturally diverse sample from Aotearoa New Zealand.

Populations of interest in science are culturally diverse. Therefore, assessing reluctance to participate in neuroimaging studies is crucial for understanding whether the findings we obtain from EEG studies are as generalizable as we hope. If some individuals of the population do not participate, whether for cultural or psychological reasons (e.g., personality traits or anxiety), and if the experimental effects are moderated by cultural or psychological characteristics, then our results will not be valid for our target population (Bulbulia 2024; Westreich et al. 2017). Moreover, if the assessment of brain activity for understanding brain mechanisms and potential treatment effects such as neurofeedback for emotion regulation training is modified by cultural values, clinical applications of EEG research will be biased (Hall et al. 2018).

Previous work finds that descriptions of neurophysiological methods can evoke both intrigue and fear (Martin and Nobar‐Nazari 2013) and such emotions in turn have been linked to skepticism (Wardlaw et al. 2011). Ostensibly, such emotions and judgments might affect recruitment, leading to selection bias (Chiao and Cheon 2010). Although researchers have long recognized that sampling is a problem for psychological science (Cheon et al. 2020; Henrich et al. 2010), the types of assessments used in neurophysiological research may amplify the problems. For example, the use of EEG to measure brain activity in the context of experimental or intervention research like neurofeedback requires placing sensors on the scalps of participants.

Apart from methodological questions related to hair and skin type (Adams et al. 2024; Choy et al. 2022; Penner et al. 2023), certain cultures prohibit head touching. For example, in Aotearoa New Zealand's Māori culture (the indigenous peoples of Aotearoa New Zealand), the head is *tapu* (sacred, prohibited, restricted, or under protection of the gods) (Hickey 2006). In that context, Elder's (2012) work on traumatic brain injury among Māori, *Tuku iho, he tapu te upoko* (from our ancestors, the head is sacred), points out the cultural significance of the head. For many Māori, what is *tapu* must be treated with respect and caution, meaning that the head is not open to uninvited touching by strangers. Although some Māori use *karakia* (ritual chants, incantations, or prayers) in medical settings to acknowledge the *tapu* nature of the head and create a culturally safe environment (Elder 2012), there is little evidence that such practices have been adopted for neurophysiological research.

Another group of people for whom EEG methods could be of concern are religious groups that use head coverings, for instance, Muslims who use hijab (Attum et al. 2023). Several studies mention participants withdrawing participation in neuroimaging research due to a reluctance to take off head coverings (Nizam et al. 2022; Safati and Hall 2019). At the same time, a lack of research with Muslim women, especially in a context where they might represent a minority, has been highlighted for mental health research in the USA (Awaad et al. 2022). Although there are some recommendations for working with Muslim women in health care and mental health research (Attum et al. 2023; Hasnain et al. 2011; Ibrahim et al. 2022), it is unclear if women wearing a head scarf are less likely to take part in neuroscientific research compared to other groups due to the involvement of the head in EEG research. To the best of our knowledge, there have not been any studies to date that explore the attitudes towards neurophysiological research among women who wear head coverings.

One application of EEG beyond experimental and observational research is neurofeedback training. EEG‐based neurofeedback is an operant‐learning‐based approach to modify brain activity. Participants or users would see an aspect of their current brain activity illustrated on a computer screen and can develop strategies to modulate that activity. The visual representation of their brain activity is used to guide that process and positively reinforce changes in the aspired direction (Huster et al. 2014). Neurofeedback training is therefore one potential context in which EEG can be used as an intervention, which even more than general research requires less biased sampling to test its effectiveness. Most widely applied in the context of treatment of psychopathological or neurodiverse conditions (Enriquez‐Geppert et al. 2017), neurofeedback training can be used to increase emotion regulation abilities (Abdian et al. 2021; Cavazza et al. 2014). The difference between neurofeedback‐based emotion regulation training and (other) cognitive trainings (Cohen and Ochsner 2018) is that the neurofeedback approach does not provide specific instructions but rather allows the user to find their own personal and most effective strategy (Enriquez‐Geppert et al. 2017). Given that neurofeedback training is a research target for emotion regulation abilities, asking about attitudes towards this method is crucial to investigate acceptance and interest in such methods and how attitudes might differ due to cultural affiliations.

To summarize, despite evidence that cultural norms may affect participation and modify results in EEG‐based research, we are unaware of any previous study that has investigated cultural inhibitions for participation in neuroscientific research. Here, we investigate attitudes towards EEG and neurofeedback training as an emotion regulation intervention among people with various cultural affiliations living in Aotearoa New Zealand. To allow an inclusive perspective on cultural affiliation, we aimed to provide a flexible way of describing one's cultural affiliation, including dimensional choices across multiple cultural groups. We took a descriptive approach, characterizing the overall sample and reporting the views of the small culturally diverse sample as a whole for two reasons. First, allowing for describing one's ethnic affiliation for several groups instead of forced choice acknowledges that people define their identity individually and not as a hereditary construct (Gaitenidis 2024; Yampolsky and Amiot 2016). Yet, where individuals choose multiple affiliations, subgroup analyses would be inappropriate as the subgroups can overlap. Second, as cultural affiliations in indigenous groups tend to be more diverse within their group, subgroup analyses would wrongly assume homogeneity within subgroups. Notably, the New Zealand census lets people tick all ethnicities that apply. In the 2018 census, 13% of respondents identified with two or more groups. In New Zealand, then, single‐group analyses inevitably misrepresent a sizeable slice of the population (Dixon et al. 2024). For this reason, health‐monitoring scholars recommend using total‐response or combination categories that respect self‐defined, fluid identities rather than forcing participants into tidy (and unrealistic) boxes (Cormack and Robson 2010; Didham and Callister 2012). As views and attitudes regarding EEG, neurofeedback, and emotion regulation can be explored better in free text entries, we predominantly used open answer questions, supplemented by some rating questions. We expected that participants would overall express interest but also concerns regarding EEG and neurofeedback.

## 2 Methods

### 2.1 Sample

The study was advertised in the community through social media, flyers, and community groups.^1^ Of the 292 participants who started the survey, 181 participants finished the survey and can be considered as this indicated that they upheld their consent to participate. Of the included 181 participants, 127 self‐identified as women, 45 as men, one as transgender, one as nonbinary, and 7 preferred not to answer this question. Participants were on average 27.95 years old (SD = 10.04, range = 18–60). 51 (29.31%) participants had a high school degree, 13 (7.47%) vocational training, 70 (40.20%) a Bachelor's degree, 28 (16.09%) a Master's degree, and four (2.29%) a Doctorate, while 8 (4.59%) had a non‐specified degree (7 participants with missing data). Of the 181 participants, 104 (59.77%) described their main current occupation as student, 29 (16.67%) as professional, seven (4.02%) as manager, four (2.30%) as technician, four (2.30%) as service and sales, and 33 (18.97%) as other areas of occupation (7 participants had missing data).

Participants also described on a scale from 1 (*very poor*) to 5 (*very good*) their web‐search skills as good (*M* = 4.32, SD = 0.81), their ability to use digital technologies as good (*M* = 4.10, SD = 0.87), their ability to use computers as good (*M* = 4.26, SD = 0.82) and their ability to use the internet as very good (*M* = 4.56, SD = 1.69). The study was approved by the Victoria University of Wellington Human Ethics Committee, and participants provided informed consent at the start of taking the survey.

### 2.2 Measures

A series of questions on attitudes towards EEG, neurofeedback, and emotion regulation were developed and piloted for semantic understanding with categorical and free text answer styles (see Table 1). Nominal items were used as categorical variables, scale items were used as linear variables. Free text items were analyzed with Content Analysis (see section below).

**TABLE 1:** Survey questions, including categorical, rating, and free text answering options.

Question	Answer style	Answer options
Have you ever heard of a neuroscience method called electroencephalography (or short EEG) before?	NOMINAL	“Yes, I know what that is.”/“Yes, I heard of it before, but I could not tell you what it means.”/“No, never heard of it before.”/“I'm not sure.”
Have you ever heard of a neuroscience method called neurofeedback before?	NOMINAL	“Yes, I know what that is.”/“Yes, I heard of it before. but I could not tell you what it means.”/“No, never heard of it before.”/“I'm not sure.”
Please describe below what your understanding of electroencephalography (EEG) is.	FREE TEXT	
Please describe below what your understanding of neurofeedback is.	FREE TEXT	
Now that you learned about neurofeedback and its applications: How COMFORTABLE you would feel about having a cap similar to this put on your head?	SCALE	“Very uncomfortable” = 1 to “Very comfortable” = 9
How POSITIVE or NEGATIVE you would feel about learning emotion regulation by the means of this device?	SCALE	“Very negative” = 1 to “Very positive” = 9
How INTERESTED you would be in improving your emotion regulation?	SCALE	“Not interested at all” = 1 to “Very interested” = 9
What are your THOUGHTS about such a method?	FREE TEXT	
Imagine you were to try out neurofeedback for yourself. What would help you to feel more comfortable in this situation?	FREE TEXT	
Does the apparent gender of the researcher (i. e. the person TOUCHING your head) matter?	NOMINAL	“Yes”/“No”/“I don't know”
Does the apparent gender of other researchers present IN THE ROOM matter?	NOMINAL	“Yes”/“No”/“I don't know”
If you are wearing a head scarf, would you be comfortable to take it off?	NOMINAL	“Only in front of people with the same (apparent) sex as me”/“Doesn't matter”/“I don't know.”/“I don't wear a head scarf.”

### 2.3 Ethnicity Affiliation

We asked participants about their primary affiliation with a cultural group with the question, “which of the following best represents your ethnic heritage?”. The options were New Zealand European (Pākehā), Māori, Pacific Islanders (e.g., Cook Islands Māori, Fiji, Tongan, Niuean, Samoan), Chinese, Indian or Not listed above. The last option allowed participants to fill in an additional heritage type. Next, we asked participants to rate on a scale from 0 (“Do not identify at all”) to 100 (“Identify fully”), how much they identify with each of the ethnic heritage groups provided. We computed a proportional score for each ethnic group variable for each participant, by summarizing the scores provided for each ethnic group per participant and dividing each ethnicity group score by that participants' sum score (see Table 2). In addition, we calculated *n* per ethnic group by coding for each participant, which ethnic groups they affiliated with at least with 1 (out of 100, “identify fully”), see last column in Table 2. With multiple ticks allowed, chosen at least with 1 out of 100, the selected ethnicities were NZ European (*n* = 114), Māori (*n* = 70), Chinese (*n* = 57), Indian (*n* = 51) and Pacific Islanders (*n* = 47), every other option drew only 1 to 17 endorsements (see Table 2). We did not stratify responses by cultural affiliation because many participants ticked several ethnicities, creating intersecting, non‐independent subgroups (Allan 2001). Comparing such small, overlapping groups would yield low statistical power and a high risk of spurious findings (Burke et al. 2015). Moreover, forcing mixed‐heritage participants into a single category can bias estimates and misrepresent their identities (Cormack and Robson 2010). We therefore collected ethnicity data solely to illustrate the breadth of affiliations in the sample, rather than to venture into under‐powered subgroup tests. Instead, asking participants to indicate their cultural affiliation was to survey the breadth of ethnic affiliation.

**TABLE 2:** Affiliation scores with ethnicities, raw and proportional for each individual.

	*M* (*M*_prop_)	SD (SD_prop_)	*n* Affiliation score > 0
Māori	17.32 (0.13)	32.09 (0.24)	70
NZ European	39.29 (0.36)	40.75 (0.39)	114
Pacific Islander	8.45 (0.06)	23.04 (0.17)	47
Chinese	11.76 (0.11)	24.67 (0.26)	57
Indian	11.84 (0.11)	28.02 (0.29)	51
African	2.62 (0.03)	15.19 (0.16)	6
North American	0.55 (0.01)	7.43 (0.07)	1
South American	1.66 (0.06)	12.80 (0.21)	13
European	5.99 (0.05)	22.74 (0.18)	13
East Asian	1.66 (0.01)	12.80 (0.09)	3
South Asian	6.01 (0.07)	22.70 (0.23)	13
Southeast Asian	8.34 (0.06)	26.50 (0.21)	17
West Asian	0.78 (0.01)	8.05 (0.08)	2

*Note:* Range for all ethnicity affiliation ratings was 0–100, *N* = 181; *M*_prop_ = Mean proportional score for each ethnic group variable for each participant (sum of scores for each ethnic group per participant divided by participants' sum score); *n* (affiliation score > 0) = number of participants per ethnic group identifying at least with 1 (out of 100, “identify fully”).

### 2.4 Procedure

The study was conducted in an online survey format, which was advertised through social media, flyers, and community groups (see details in sample description). Once participants gave informed consent, they were asked about their ethnic affiliation. Next, they were asked if they had heard about EEG and neurofeedback before and were then shown short descriptions with images explaining how EEG and neurofeedback work. Participants were then asked the remaining attitude questions about EEG, neurofeedback, and emotion regulation interventions. At the end, participants were asked demographic and computer skills questions. Finally, they received a coffee voucher (worth NZD 4).

### 2.5 Statistical Analyses

Descriptive statistics are provided for all scale‐based questions. Correlation coefficients and regression analysis were used to compare and quantify the relationship between the responses with dimensional answer styles.

### 2.6 Content Analyses

A content analysis was conducted on the responses of the 181 participants' answers to six open‐ended questions, using NVivo coding software (version 14). Codes were generated inductively from the data, whereby participants' responses were analyzed for the key meaning of the text. Participants' responses were coded either as whole texts or were separated if there were multiple ideas in a response. For example, if a participant described EEG as being “too scary” this was coded as one idea, but if a participant described EEG as being “too scary and untrustworthy” this was coded as two separate ideas. Ten percent of the data were coded by a second coder to assess inter‐rater reliability. Following this, a comparison query was run in NVivo, and interrater reliability (Kappa) was computed for each question, ranging between *κ* = 0–1 (Mean *κ* = 0.71). Given a Kappa value of 0 indicates chance agreement and 1 indicates perfect agreement, the average Kappa for the current research indicates good agreement (Gisev et al. 2013).

## 3 Results

### 3.1 General Knowledge About EEG

For the question: “Have you ever heard of a neuroscience method called electroencephalography (or short EEG) before?”, 29.28% of the 181 participants knew what EEG is, 23.76% had heard about it but did not know what it is and 35.91% had not heard about it, while 11.05% were not sure.

In the related free text question, “Please describe below what your understanding of electroencephalography (EEG) is.”, participants most commonly reported that they did not know what EEG was (56 occurrences). The second most common response was that EEG measures brain activity (37 occurrences), followed by it being something that detects electrical activity in the brain (29 occurrences). Some participants noted that it used electrodes (27 occurrences), that it measures brain waves (19 occurrences), and that it involves something about the brain (16 occurrences). Less common responses were that measurement occurs on the head (9 occurrences), that it is used for diagnosis (8 occurrences), that it measures neurons (5 occurrences), that it has something to do with physiological activity (5 occurrences), that it is a brain scan (5 occurrences) and that it detects/measures brain cell movement and function (3 occurrences). The dataset contained 12 other codes that only had one occurrence each for this question. Overall, the knowledge level about EEG was rather low, and participants described it by use of electrodes and involves (electrical activity in) the brain (see Table 3).

**TABLE 3:** Response codes from answers for general knowledge about EEG.

Code	Number of occurrences	Example response
Don't know	56 (23.1%)	“I don't have an understanding of it”
Measures brain activity	37 (15.3%)	“It's measuring electrical activity on your brain, and you put it on your head (scalp).”
Detects electrical activity in the brain	29 (12.0%)	“It's measuring electrical activity on your brain”
Uses electrodes	27 (11.2%)	“Using a cap with electrodes”
Measures brain waves	19 (7.9%)	“Something that reads brain waves”
Something about the brain	16 (6.6%)	“I think it has something to do with brain measurements”
Measurement occurs on head	9 (3.7%)	“Something you put on your head”
Used for diagnosis	8 (3.3%)	“I think EEG is a test that records brain activity and is used to help the diagnosis of the brain's condition.”
Measures neurons	5 (2.1%)	“Test that detects neuron activities in the brain”
Brain scan	5 (2.1%)	“Brain scan – images activity?”
Physiological activity	5 (2.1%)	“Detect physiological activity”
Braincell movement and function	3 (1.2%)	“Examine the activation or function of brain cells.”
No response	11 (4.5%)	

*Note:* Other mentions (1 each): Electric shock therapy, Electronic images, Emotional control, Graphing using electronics, Measures brain functions, Measures how brains process information, Measures reaction times, Monitors face movement, Recorded in waves, Related to electrons, Test for something, Used to find differences in people.

### 3.2 General Knowledge About Neurofeedback

When asked if they had heard of neurofeedback before, 7.73% of participants stated that they knew what it was, 21.55% had heard about it but did not know what it was, and 45.30% had not heard about it, while 23.76% were not sure.

For the related free text question, “Please describe below what your understanding of neurofeedback is”, the most common response was that it involved feedback from brain waves and/or activity (7 occurrences), followed by that it is used to re‐train brain responses (6 occurrences), that it uses EEG (5 occurrences) and that it is used for self‐regulation (3 occurrences). The dataset contained 7 other codes that only had one occurrence each for this question (see Table 4).

**TABLE 4:** Responses for general knowledge about neurofeedback.

Codes	Number of occurrences	Example response
Feedback from brain waves/activity	7 (3.6%)	“Feedback of your brain activity from a computer program?”
Re‐train brain responses	6 (3.1%)	“Re‐train to more appropriate responses”
Uses EEG	5 (2.6%)	“Neurofeedback is kind of brain training, which could use a real‐time EEG pattern to help people act more appropriately by observing the brain wave patterns.”
For self‐regulation	3 (1.6%)	“Neurofeedback is biofeedback which helps brain to self‐regulate”
No response	165 (85.5%)	

*Note:* Other mentions (1 each): Biofeedback, Influence brain activity, Nervous system response, Neuron inputs, Recordings of brain activity, See brain activity, Uses brain scan.

### 3.3 The Assessment Environment

When asked how important the gender of the experimenter touching their head would be in the context of participating in a study involving EEG measurement, 19.34% of participants indicated that the gender of the experimenter would matter for them, while 71.27% indicated that it would not matter, and 5.52% indicated that they did not know if it would matter. The gender of experimenters present in the room was indicated as relevant by 17.13%, but as not relevant by 71.27% of participants and unknown if it would matter for them by 7.73%. The importance of the gender of the experimenter and the experimenters present in the room significantly correlated (*r* = 0.51, *p* < 0.001). Of participants wearing a head scarf (12.70%), 30.43% indicated they would only take off their head scarf in front of people of the same sex, while 52.17% indicated that the gender of those around them would not matter, and 17.39% indicated that they were unsure whether it mattered to them.

When asked, “Imagine you were to try out neurofeedback for yourself. What would help you to feel more comfortable in this situation?”, participants noted clear and informative explanation and instructions most commonly (37 occurrences), followed by a relaxed and supportive environment (21 occurrences) and an environment that has privacy and quiet (21 occurrences). Participants also reported that comfortable equipment (20 occurrences), an empathetic, safe, and knowledgeable person administering the equipment (18 occurrences), and the presence of a friend or family/whānau member (15 occurrences) would make them more comfortable. Safety and the reassurance that this process would not have any negative side effects was also reported (14 occurrences), as was a physically comfortable and relaxed space (12 occurrences). Some participants did not know what would make neurofeedback more comfortable (7 occurrences), and others expressed that there was nothing that would make them more comfortable (5 occurrences). Some participants expressed that they would need to focus on the benefits from engaging in this method (5 occurrences). Other suggestions that participants indicated to make them more comfortable with 4 occurrences each were: a video showing the neurofeedback process, not doing it at all, giving informed consent (e.g., only participating when they were ready, being able to withdraw at any time), and having information about other studies that used this method. Participants also suggested that having a test session before hand, there being food/drink available, and doing it at home would make them more comfortable (3 occurrences each). The dataset contained 18 other codes that only had one or two occurrences each for this question (see Table 5).

**TABLE 5:** Example responses for what would make participants more comfortable.

Codes	Number of occurrences	Example response
Clear and informative explanation and instructions	37 (14.8%)	“An explanation without jargon”
Relaxed and supportive environment	21 (8.4%)	“Relaxed surroundings, informal setting”
Privacy and quiet	21 (8.4%)	“Doing the activity alone”
Comfortable equipment	20 (8.0%)	“A more comfortable cap”
Empathetic, safe, and knowledgeable person	18 (7.2%)	“Someone friendly in the room with me to help me relax a little”
Presence of a friend or family/whānau member	15 (6.0%)	“Having someone I know”
Safety ensured, reassurance that this process would not have any negative side effects	14 (5.6%)	“Knowing that it is not physically harmful”
Physically comfortable and relaxed space	12 (4.8%)	“Being in a comfortable room”
Don't know	7 (2.8%)	“Not sure”
Nothing	5 (2.0%)	“Nothing personally…”
Video showing the neurofeedback process	4 (1.6%)	“Seeing a short video outlining the procedure”
Not doing it at all	4 (1.6%)	“Not doing it would make me feel most comfortable!”
Giving informed consent	4 (1.6%)	“The ability to stop anytime”
Having information about other studies that used this method	4 (1.6%)	“More information about other studies”
Test session before hand	3 (1.2%)	“A short session to warm up to it with a break and then a proper session.”
Food/drink available	3 (1.2%)	“Snacks and drink available”
Doing it at home	3 (1.2%)	“Ability to run the process in own home or neutral space”
No response	20 (8.0%)	

*Note:* Other types of mentions (1–2 each): Already comfortable, Clinical setting, Data not available to others, Emphasis on it being only one skill, In a group setting, Show how it works first, Simple process, Slow‐paced process, Able to ask questions throughout, Able to get comfortable with equipment, Calming music, Chance to see the space prior to experience, Consent to touch head, Cultural understanding, Distractions, Multiple opportunities, Person administering rather than computer, Shared culture with person administering.

### 3.4 About Using Neurofeedback and Training Emotion Regulation

**FIGURE 1:**
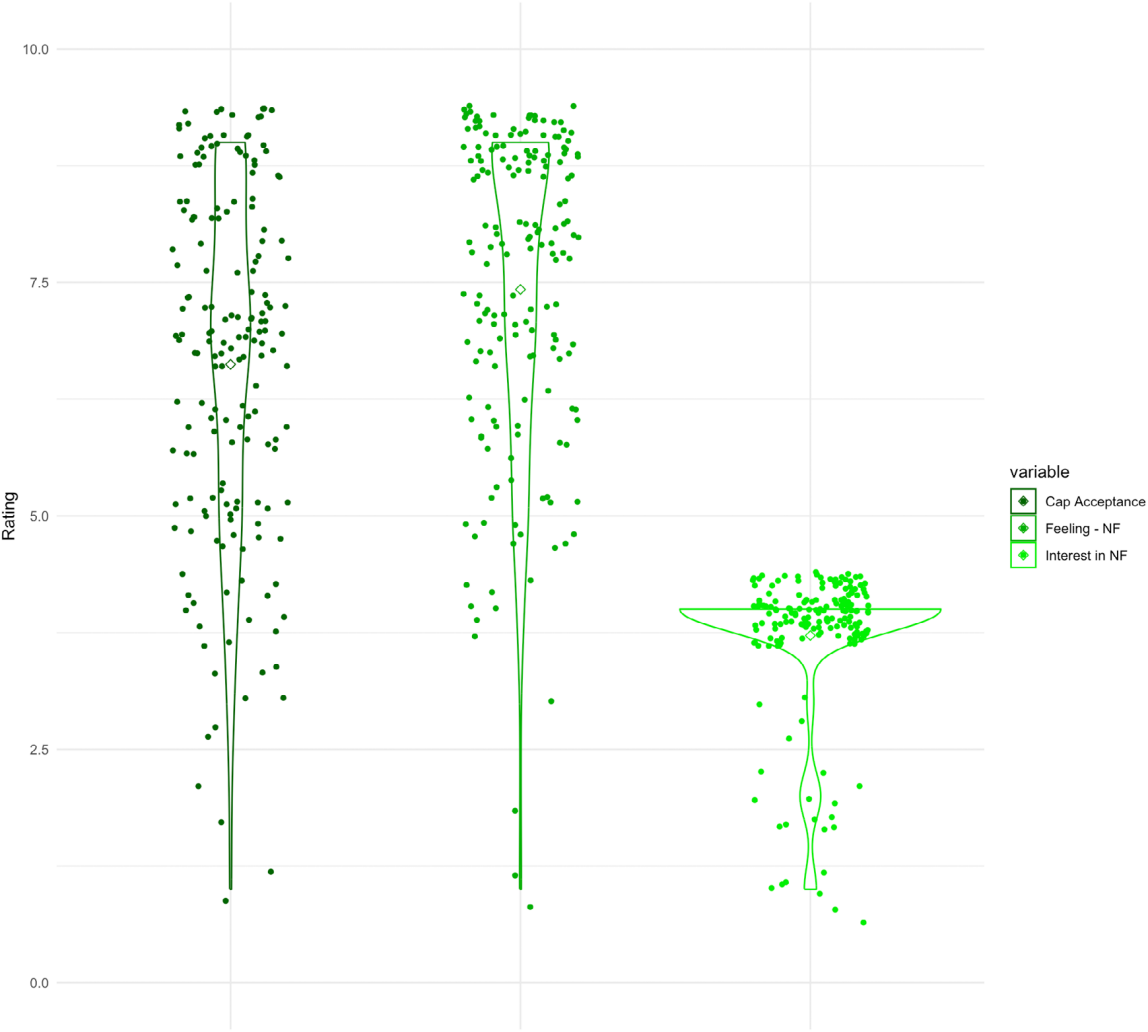
Individuals' ratings for being comfortable with wearing an EEG cap on the head (Cap acceptance), feeling about neurofeedback as an emotion regulation training method (Feeling—NF) and interest in improving emotion regulation through neurofeedback (Interest in NF). Rating scale ranging from 1 to 10.

Overall, participants described they would feel rather positive about using neurofeedback as a method to improve emotion regulation (*M* = 6.62, SD = 1.88, range = 1–9, on a scale from 1 = *very negative* to 9 = *very positive*), are rather interested in improving their emotion regulation abilities (*M* = 7.43, SD = 1.75, range = 1–9, on a scale from 1 = *not at all* to 9 = *very interested*), and would be moderately comfortable to wear an EEG cap after they learned about its function during the survey (*M* = 6.53, SD = 2.22, range = 1–9, on a scale from 1 = *not at all* to 9 = *very comfortable*), see Figure 1.

The ratings were all correlated: Participants who felt more comfortable wearing an EEG cap felt also more positive about using neurofeedback as an emotion regulation training tool (*r* = 0.59, *p* < 0.001) and were more interested in trying neurofeedback (*r* = 0.39, *p* < 0.001). Those who felt more positive about using neurofeedback were also more interested in trying it (*r* = 0.68, *p* < 0.001). To better understand which variables contribute most to an interest in taking part in EEG and neurofeedback research, we ran a multiple linear regression model with the outcome variable how comfortable participants would feel with wearing an EEG cap and the predictors existing basic knowledge about EEG, existing knowledge about neurofeedback, how they feel about neurofeedback as an emotion regulation training method, and Interest in improving emotion regulation with neurofeedback. This resulted in a statistically significant model, *F*(4,169) = 29.97, *p* < 0.001, *R* ^*2*^ = 0.40, with only basic knowledge about EEG (*b* = 0.99, *p* < 0.001) and feelings about emotion regulation (*b* = 0.65, *p* < 0.001) positively contributing to the model.

When answering the question, “What are your THOUGHTS about such a method [emotion regulation]?”, participants most commonly reported that they found this process interesting (42 occurrences), that they thought it would be helpful for mental health and wellbeing (23 occurrences), and that they were curious to try it (19 occurrences). Following this, participants reported that it seemed like a good and effective idea (15 occurrences), although some participants reported that they did not trust this method (14 occurrences). Participants also noted that it would be a good method to understand thoughts, actions, and feelings (13 occurrences), whereas some reported that they did not know enough about it to make a statement (10 occurrences). Less commonly, participants reported that they thought it could change/improve someone's life (7 occurrences), that it seems scary (7 occurrences), that it feels invasive and too science‐y (7 occurrences), and that they would be worried about side effects (6 occurrences). Some participants stated that they would be uncomfortable with this process (5 occurrences) and that they would prefer counseling (4 occurrences). Comparatively, some participants reported that they thought it was not intrusive (4 occurrences), whereas others believed it was not culturally sensitive or aligned with Tikanga Māori (4 occurrences), and that it was expensive and inaccessible (4 occurrences). The dataset contained 31 other codes that only had one or two occurrences each for this question (Table 6).

**TABLE 6:** Example responses for the use of neurofeedback for emotion regulation training.

Codes	Number of occurrences	Example response
Interesting process	37 (15.0%)	“A very interesting way to understand how your brain works”
Helpful for health and wellbeing	23 (9.3%)	“Has some important uses regarding mental health”
Curious to try	19 (7.7%)	“I would like to try it”
Good and effective idea	15 (6.1%)	“I think this method is probably the most effective method”
Do not trust this method	14 (5.7%)	“I don't know if I would trust it”
Good method to understand thoughts, actions, and feelings	13 (5.3%)	“It could help me understand my emotions and control it better”
Don't know enough	10 (4.1%)	“Would need to read more related research”
Could change/improve someone's life	7 (2.8%)	“it can improve lifestyle”
Seems scary	7 (2.8%)	“Looks scary”
Feels invasive and too science‐y	7 (2.8%)	“I feel it is highly invasive”
Worried about side effects	6 (2.4%)	“I'm a bit skeptical about how it would work and any side effects”
Would be uncomfortable	5 (2.0%)	“Personally I would not be comfortable…”
Would prefer counseling	4 (1.6%)	“I would prefer something like counseling if I needed to regulate my emotions”
Not intrusive	4 (1.6%)	“It seems non‐intrusive and low stakes”
Not culturally sensitive or aligned with Tikanga Māori	4 (1.6%)	“Being Māori ‐ the head is considered tapu so having something like this on can be interpreted by some as too invasive.”
Expensive and inaccessible	4 (1.6%)	“I think that it could be useful – but an expensive form of therapy”
No response	37 (15.0%)	

*Note:* Other types of mentions (1–2 each): Bad idea, Benefits outweigh the potential discomfort, Boring, Can identify problems, Conspiracy beliefs, Could change personality, Curious about how else it could be applied, Don't need it, Don't understand, Easy experience, Good way to see the truth, Hard to convince people to participate, Moral and ethical concerns, More engaging method than therapy, More legitimate than other methods, Not effective, Painful, Preferred method, Similar to meditation, Time consuming, Unhelpful, Unsure, Would not want to try, Can treat addictions, Concern about data security, Concern about potential judgments and stereotypes, No thoughts, Results are informative, Should not be widely available, Similar effects could be gained differently, Treatment doesn't seem realistic, Want to know more.

## 4 Discussion

This study investigated perceptions of Electroencephalography (EEG) and neurofeedback methods and suggestions about what would make participating in this type of research more comfortable in a culturally diverse group from Aotearoa New Zealand. Content analyses of the open‐text answers and descriptive statistics across the diverse sample showed initially that most participants were not sure what EEG was or were unsure about its function. Overall, based on responses to individual questions (but not based on their self‐reported understanding from the Likert scale measures) participants tended to descriptively group into three qualitative categories: those that had knowledge about EEG and neurofeedback and were comfortable with these methods, those that did not have knowledge about them, but would be willing to learn more and potentially try them if they had more information, and those that did not have knowledge about them but did not trust the methods so would not be willing to try them. For those that did not have knowledge about the methods, it appeared that the main difference between those who were willing to learn more and potentially try them, and those that were not, was their overall trust in science. For participants that were unwilling, they were cautious of the potential harm that could result from these methods, and thus believed engaging in them was not worth the risk. Negative views and cautiousness were rare, and most of the codes derived from the question about trying the method were positive, which might be due to the sample already interested in research and therefore participating in the current study. However, for participants that were open to the methods, they expressed a desire for clear and accessible information provided by a friendly and knowledgeable professional to build the trust needed for their engagement. Thus, a vital aspect of making people more comfortable with methods like EEG and neurofeedback is to bridge the gap between science seeming far‐fetched, inaccessible, and ‘alien’, as some participants described, and it being a collaborative process between researchers and study participants.

Interestingly, the overall willingness to try neurofeedback as a tool to improve emotion regulation was unexpectedly positive. Although some participants did raise concerns and were more reluctant to engage in EEG‐based neurofeedback, most of the diverse sample of participants were interested in participating and envisioned benefits for wellbeing and health. This is encouraging, as it could imply that—given that potential participants are provided with detailed information in the recruitment context to allow them to decide on the basis of more accessible information than most recruitment information might be—more diverse samples could be included in EEG and neurofeedback research.

A low knowledge base in a general population sample about what EEG is and how it can be used is not necessarily astonishing, given that these are not very commonly used methods. Our study results underline not only that this is the case but also that participants indicate that having more information about the method, its effects, and usage would be necessary to make them feel comfortable with taking part in such research. This aligns with findings that knowledge can increase attitude‐behavior consistency (Fabrigar et al. 2006). Therefore, providing more information in adequate ways (e.g., providing an educational video about what taking part in such a study would look like as one of our participants suggested) could address hesitations due to little knowledge. This could not only increase recruitment into EEG‐based research but could also improve participant retention in such research.

In our view as neuroscientists with applied interests, it is encouraging that there is openness to EEG and neurofeedback across a culturally diverse sample, including individuals with potentially varying cultural norms that involve wearing a hijab or consider the head as sacred, which contrasts the concerns we suggested at the outset of the current study. Also encouraging, responses reveal potential to address sampling bias in neurophysiological studies by creating a safe and culturally responsive space through the practices suggested by the participants. These include good information about the methods and instruments used before the consent process, a continual process for informed consent, the clear communication of choice as well as a comfortable environment. This has been showcased for the Aotearoa New Zealand context with the specific spiritual relevance of the head. The free text answers showed that diverse cultural affiliations might bring concerns and beliefs with them that might conflict with measures such as EEG, which have not been considered so far in neuroscientific research. Thus, through this study, we were able to derive valuable results that might help to identify general hesitations and needs of potential participants to sign up for neuroscientific research.

Our study has several limitations. First, we could not gauge the views of those who declined, or would likely decline, an EEG‐recruitment survey, so the direction and size of any non‐response bias remain unknown. Every study must specify a target population, and here our sample simply offers a glimpse of the diversity available to the population of online volunteers (Bulbulia 2024). Second, participation required some interest in brain research and hence self‐selection almost certainly shaped our cohort. We did not test associations between ethnicity and willingness to join neuroscience studies because many participants identified with multiple ethnicities, creating small, overlapping, non‐independent subgroups. Larger, purpose‐built samples might investigate such associations in a broader population. Third, despite wide advertising, our respondents were comparatively well‐educated, so the findings may under‐represent skeptical or resource‐constrained communities. Future work might examine how personality, education, religious beliefs, and cultural norms jointly influence both recruitment and retention in EEG projects. Finally, because we allowed nuanced, multi‐ethnic self‐identification (Gaitenidis 2024; Yampolsky and Amiot 2016) but chose not to slice the data into overlapping fragments, our results reflect the aggregate picture rather than the fine‐grained concerns of any single group. Qualitative work targeted at specific communities could fill that gap. In short, these preliminary findings highlight recruitment and retention challenges for EEG (and especially neurofeedback) research. We hope they inspire richer investigations into the cultural, educational, and attitudinal filters that still keep many would‐be participants on the sidelines. We conclude with five practical suggestions, based on the insights reported above, which may improve the current state of practice.

### 4.1 Implications for an Inclusive EEG‐Based Neuroscience Research Practice

Based on our findings and the suggestions mentioned by the participants in our study, there is the opportunity to derive some implications for research practice. These implications will help investigators to develop protocols that improve upon their current designs. Importantly, ethnic affiliation information should not be used as a basis for exclusion. Nor should any variables affected by the treatment be included in statistical models for its effects on experimental outcomes (Bulbulia 2024; Montgomery et al. 2018). In addition, considerations of biases in the uptake of research participation can contribute to the awareness of EEG research and its utility in the general population. Therefore, we derived the following suggestions for improving recruitment and the research environment, grouped into five simple steps for more broadly inclusive EEG‐based research:

First, advertise studies implying the option to have only a female researcher present: When recruiting participants for a study that includes EEG assessment, researchers should make potential participants aware that they can indicate if they would prefer a male or female researcher present, or if they do not mind.

Second, share as much information about the steps of the study procedure in advance as possible: During the recruitment process, try to include as much information about the process and what participants have to expect during the assessments, including which body parts are going to be involved and how, as these processes might be completely new to them and difficult to imagine. For example, an informative video that shows what it looks like to be a participant in such a study could be shared (Example video can be obtained from the corresponding author), or images and short descriptions that allow one to imagine the process.

Third, provide a comfortable, inviting space: Research facilities often are either very scarce and simple or overloaded with technical equipment. Preparing the rooms that participants will enter to be professional, but also inviting and comfortable (e.g., by using comfortable chairs and an inviting interior). This atmosphere can reduce participant stress and enhance the quality of neurophysiological data collected. An inviting space may also improve participant retention and willingness to engage in future research sessions.

Fourth, explain the procedure step‐by‐step at the start and throughout the assessment: The researcher should explain every step of the procedure before asking for consent. This initial explanation helps to build trust and ensures that participants are fully aware of what to expect, reducing anxiety and increasing compliance. Throughout the assessment, researchers should ask for consent for every step of the process, ensuring that participants feel in control and respected. Reiterating consent reinforces the participant's autonomy and can enhance their comfort and cooperation. Additionally, this continuous communication allows participants to ask questions and express concerns, which can be addressed immediately, thereby improving the overall quality of the subjective research experience. This meticulous approach to consent can lead to more accurate and reliable data collection.

Fifth, provide opportunity for feedback: At the end of the participation, allow time and space for participants to share their feedback about taking part in the research. This might involve putting time aside for verbal feedback or written (anonymous) feedback through a feedback survey form. Verbal feedback can offer immediate and detailed responses, whereas anonymous written feedback can encourage honesty and openness. Gathering feedback is crucial as it provides insights into the participants' experience, highlighting any areas that may need improvement. This process not only helps researchers refine their procedures and enhance future studies but also shows participants that their opinions are valued and can improve participant retention.

## 5 Conclusion

This study derived several important ideas and suggestions to consider in the context of EEG research in terms of recruitment and procedures during such research to be more inclusive. Although the participants in the current study might not be representative of the general population, the variety in cultural affiliations allows us to draw on a wider community view about EEG research and to derive some clear practical considerations. This should be a starting point for further investigation and consultation with potential research participants to increase awareness of EEG research and long‐term reduce bias in EEG research samples.

## Author Contributions

**Hedwig Eisenbarth:** conceptualization, formal analysis, methodology, project administration, resources, supervision, validation, writing – original draft, writing – review and editing. **Chelsea D'Cruz:** formal analysis, writing – review and editing. **Joseph A. Bulbulia:** methodology, writing – review and editing. **Bohemian Thanni:** formal analysis, validation.

## Conflicts of Interest

The authors declare no conflicts of interest.

## Data Availability

Data are available from the authors on request.

## Endnotes

^1^
Flyer text: “Would you like to help us understand what people from different cultures think about brain trainings? Share your thoughts on your culture and brain research. Research has been focusing on Pākeha/NZ European people, but we want to hear different cultures' voices.”
